# A simple, low cost GC/MS method for the sub-nanogram per litre measurement of organotins in coastal water

**DOI:** 10.1016/j.mex.2016.07.001

**Published:** 2016-07-20

**Authors:** Russell F. Cole, Graham A. Mills, Adil Bakir, Ian Townsend, Anthony Gravell, Gary R. Fones

**Affiliations:** a School of Earth and Environmental Sciences, University of Portsmouth, Burnaby Building, Burnaby Road, Portsmouth PO1 3QL, UK; b School of Pharmacy and Biomedical Sciences, University of Portsmouth, St. Michael’s Building, White Swan Road, Portsmouth PO1 2DT, UK; c South West Water Ltd., Bridge Road, Countess Wear, Exeter EX2 7AA, UK; d Natural Resources Wales, Llanelli Laboratory, 19 Penyfai Lane, Furnace, Llanelli SA15 4EL, UK

**Keywords:** Measurement of organotins in coastal water using GC/MS, Organotins, Tributyltin, Coastal water, Gas chromatography/mass spectrometry, Large volume injection, Liquid-liquid extraction, Ethylation

## Abstract

Tributyltin (TBT) is a legacy pollutant in the aquatic environment, predominantly from its use in anti-foulant paints and is listed as a priority hazardous substance in the European Union’s Water Framework Directive (WFD). Measuring low concentrations of TBT and other organotins (e.g. monobutyltin (MBT), dibutyltin (DBT), diphenyltin (DPhT) and triphenyltin (TPhT)) at sub ng/L concentrations in coastal waters using standard laboratory instrumentation is very challenging. Conventional, low injection volume gas chromatography/mass spectrometry (GC/MS) combined with liquid-liquid extraction typically achieves limits of detection for TBT ∼10 ng L^−1^. We describe a simple, programmed temperature vaporisation-large injection volume (50 μL), GC/MS selected ion monitoring method for measuring DBT, TBT, DPhT and TPhT in coastal waters at lower concentrations. Quantification of MBT was not possible using these injection volumes but was achieved using a 10 μL injection volume together with a reduced injection speed.

This new approach offers:

• When using a 50 μL injection, limits of detection = 0.70 ng L^−1^ and limits of quantification = 2.1 ng L^−1^ for TBT were achieved in derivatised standards.
• Recoveries of TBT and TPhT from coastal water >97%.
• Time consuming, off-line sample pre-concentration methods are unnecessary.

## Graphical abstract

**Figure:**
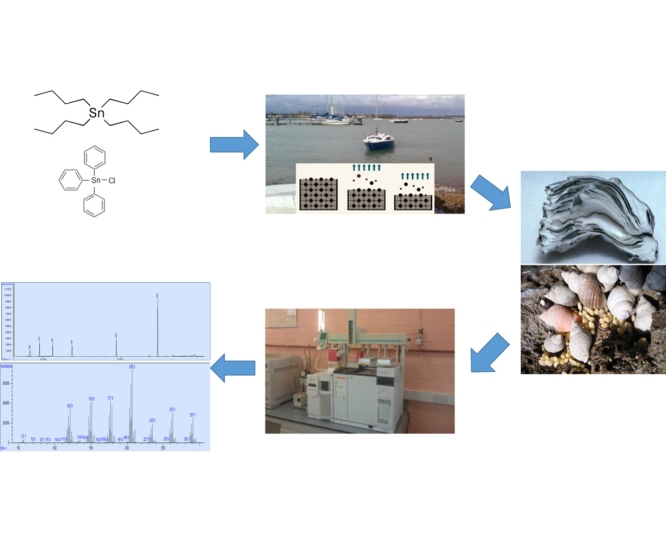

## Method details

### Safety protocol

Organotin compounds are toxic and harmful to the environment, requiring care in use [1]. Sodium tetraethylborate (NaBEt_4_) is spontaneously flammable in air and produces toxic fumes when added to water. Great care must be exercised when using these compounds and adequate control measures put in place to manage risks before performing the method described in this article. For example: (1) Standards and solutions must be handled in a fume hood fitted with a carbon filter. (2) Purchasing small quantities (∼1 g) of NaBEt_4_, negating the requirement for weighing out and reducing its exposure time to air. (3) Waste stock standards (as non-derivatised analogues) disposed of as ‘chlorinated organotin waste’.

### Preparation of glassware

Glassware is treated using the procedure described in Ref. [2]. Briefly, glassware is cleaned using a 10% Decon-90 solution at 85° C (Decon Laboratories Ltd., Hove, UK), followed by soaking in hydrochloric acid (HCl, 12 M) for 24 h. Afterwards, glassware is rinsed with water, then methanol and dried (60° C). This procedure gives low procedural blanks for all organotins measured.

### Reagents and standards

Chemicals (analytical grade or better) are from Fisher Scientific Ltd. (Loughborough, UK) unless specified. Deionised water (>15 MΩ cm, Purite Ltd., Thame, UK) is used as the laboratory water. Salts of the organotins (butyltin trichloride 97%, dibutlytin dichloride 97%, tributyltin chloride 95%, diphenyltin dichloride 98%, triphenyltin chloride 95%) are used to make stock standards (1 g L^−1^) in methanol (LC–MS Chromasolv^®^, Sigma-Aldrich, Poole, UK) with storage and expiry dates as per manufacturer’s instructions. From these an intermediate stock solution is prepared at 1.0 mg L^−1^ and further diluted to give a working standard solution of 0.05 mg L^−1^ (as organotin cation equivalents). Tripropyltin (TPrT) chloride (2.0 mg mL^−1^ in dichloromethane) internal standard is diluted in methanol (1.0 mg L^−1^, as cation equivalent). Standards are stored in amber vials at 4° C and sealed with foil-lined caps and are stable for 6 months under these conditions. Sodium acetate buffer solution (1 M, pH 4.20 ± 0.1) is used to control the pH of samples during derivatisation. Here, 136 g of sodium acetate trihydrate is added to a volumetric flask (1 L) and dissolved in water (500 mL). Glacial acetic acid (200 mL) is added slowly and then the solution diluted to volume using water. The buffer solution is stable for 6 months when stored at room temperature.

Organotins are ethylated using 1% (*m/v*) NaBEt_4_. In a fume hood, a vial of NaBEt_4_ (1 g, 97%) is filled to the neck with water and the slurry rinsed (4–5 separate washings) into a volumetric flask (100 mL) to ensure the NaBEt_4_ is completely dissolved. The solution is then diluted to volume using water (100 mL). Solutions of 1% NaBEt_4_ are stored at −20° C and are stable for up to 2 weeks.

### Sampling

Coastal water samples are collected in clean borosilicate glass bottles (1 L), sealed with aluminium foil-lined caps and are then transported to the laboratory in cool boxes [3]. Samples not analysed immediately are kept in the dark at 4° C until analysis (within 14 days).

### Extraction and derivatisation of standards and coastal water

Calibration standards are made up in volumetric flasks (250 mL) on the day of analysis. Standards (250 mL) and coastal water samples (250 mL) are analysed in the same manner using the following procedure:

1. Add 20 μL of internal standard (TPrT, 1.0 mg L^−1^) and the appropriate volume of calibration standard to a labelled 250 mL volumetric flask.
2. Using a glass funnel, add deionised/coastal water to the volumetric flask and make up to the mark.
3. Add *n-*hexane (2.0 mL) and shake by hand (∼1 min) to mix the two phases.
4. Place flask on a mechanical shaker for 15 min at 400 oscillations min^−1^.
5. Allow the *n-*hexane/water phases to separate (∼5 min). Add sodium acetate buffer solution (1.0 mL) to flask and invert to mix.
6. In a fume hood, add 1% NaBEt_4_ (1.0 mL) to flask, stopper and manually shake for 1 min. Place flask on a shaker for 10 min (400 oscillations min^−1^) to ethylate the organotin compounds.
7. Allow the layers to separate (∼30 min). Transfer carefully the *n-*hexane layer to a glass vial (10 mL). Add ∼4 g of anhydrous sodium sulphate to the vial, seal with a foil-lined cap and shake to remove any residual water in the sample. Transfer the extract into an amber vial (2 mL) for instrumental analysis.

Derivatised extracts not analysed immediately are stored at −20° C in the dark for no longer than 1 week.

### GC/MS and large volume injection conditions

**Fig. 1:**
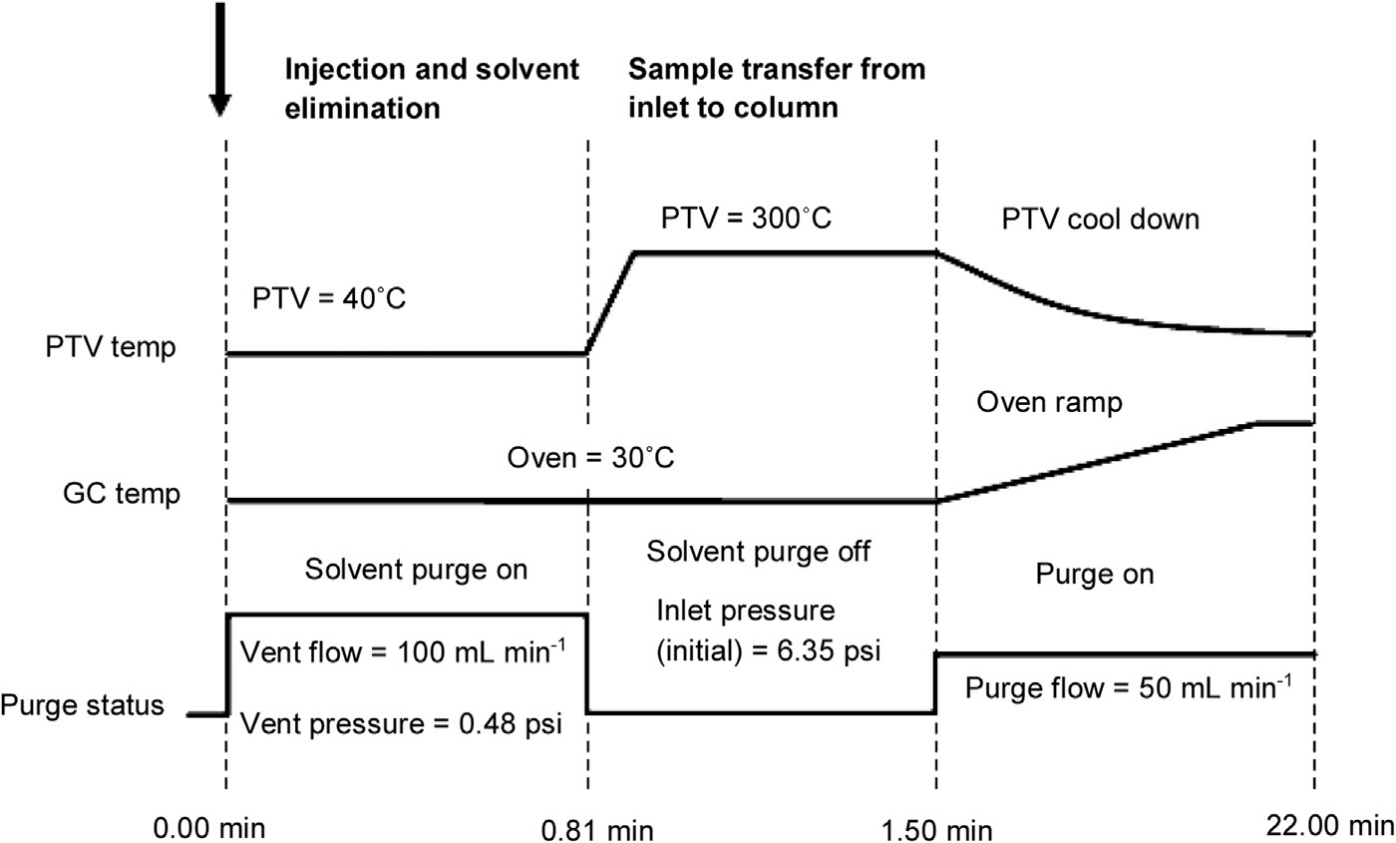
PTV-LVI temperature and flow programmes used for analysis organotins.

An Agilent 7890A/5975C GC/MS (Agilent Technologies, Santa Clara, USA) with a programmed temperature vaporisation-large volume injector (PTV-LVI) and a CTC-PAL auto-sampler (CTC Analytics AG, Zwingen, Switzerland) is used for all analyses. The SKY™ Liner, baffled PTV (1.5 mm × 3.0 mm × 71 mm, Cat. No. 23433.10) is from Thames Restek (Saunderton, UK). The GC guard column (Rxi^®^ Guard Column (Cat. No. 10029), Thames Restek) is attached to the analytical column (HP5-MS (Cat. No. 19091S-233), Agilent Technologies Ltd, Stockport, UK) using a Universal press-tight glass connector (Cat. No. 20400) (Thames Restek). Table 1 and Fig. 1 show the instrument conditions and timing intervals of the PTV-LVI programme (with flow and temperature settings adapted from reference [4]).

**Table 1:** GC/MS conditions used for the analysis of organotin compounds.

Instrument conditions	
Temperatures	
Injector temperature programme	40° C, held for 1 min
Ramp 1	300° C at a rate of 600° C min^−1^, held for 1.9 min
Ramp 2	200° C at a rate of −10° C min^−1^, held for 1 min
Final	Inlet cooled to 40° C
Oven programme	35° C for 1.5 min
Ramp 1	15° C min^−1^ to 300° C (hold 2 min)
MS interface	280° C

Injection conditions	
Injector	PTV-LVI
Syringe volume Injection volume	100 μL 50 μL
Injection speed	1 μL s^−1^

Flow rates	
Carrier gas	Helium
Injector mode	PTV solvent vent
Purge flow to split vent	50 mL min^−1^
Vent	100 mL min^−1^
Inlet pressure	0.479 psi until 0.81 min
Gas saver	20 mL min^−1^ after 2 min
Column flow rate	2.5 mL min^−1^

Columns	
Guard column	Non-polar Rxi^®^ retention gap (1 m × 0.25 mm i.d.)
Analytical column	HP5-MS (30 m × 0.25 mm × 0.25 μm film thickness)

Mass spectrometer conditions	
Mode	Electron ionisation (70 eV)
Solvent delay	8 min
EMV mode	Relative
Acquisition mode	Selected ion monitoring
Dwell time	20 (ms per mass)

(Adapted from Ref. [4]).

### Data acquisition

Data acquisition is undertaken with the MS in electron ionisation mode (70 eV) using selected ion monitoring (SIM). Quantification and confirmation ions used are shown in Table 2.

**Table 2:** SIM acquisition parameters used for ethylated organotins.

Ethylated organotin compound	Parent organotin compound	SIM group start time (min)	Quantification ion (*m*/*z*)	Confirmation ion (*m*/*z*)
Tripropylethyl-tin (ITSD)	TPrT	9.6	193	235
Butyltriethyl-tin	MBT	8.0	162	179
Dibutyldiethyl-tin	DBT	10.5	261	263
Tributylethyl-tin	TBT	11.7	289	291
Diphenyldiethyl-tin	DPhT	14.5	301	303
Triphenylethyl-tin	TPhT	16.0	349	351

ITSD = internal standard.

### Validation

External calibration is undertaken using 0, 2, 4, 8, 16, 32, 64, 80 ng L^−1^ standards; with the low calibration range matching concentrations of organotins found typically in coastal waters. The external calibration solutions are prepared as follows. Appropriate amounts of the working standard solution (0.05 mg L^−1^, see above) together with internal standard solution (20 μL, to give a concentration of TPrT = 80 ng L^−1^) are added to a 250 mL volumetric flask and diluted to volume using water. Organotins in the calibration solutions are pre-concentrated in *n*-hexane (2 mL) and derivatised using the procedure as above. Linear regression of the internally standardised calibration plot typically gives correlation coefficients (R^2^) of MBT = 0.947, DBT = 0.993, TBT = 0.998, DPhT = 0.992 and TPhT = 0.993.

Limits of detection (LoD) and limits of quantification (LoQ) are calculated using the International Conference on Harmonisation method [5]; where the standard deviation of the instrument response of the lowest calibration standard (σ) is divided by the slope (s) of the calibration curve (where LoD = 3.3σ/s, LoQ = 10σ/s). LoD and LoQ are shown in Table 3. Recoveries of organotins are derived by spiking aliquots of unfiltered coastal water (250 mL, collected from Langstone Harbour, Portsmouth, UK, on 12th February 2016) with 10 ng (≡ 40 ng L^−1^) of each compound (n = 5). The natural concentration of organotins in the sample of coastal water are given in Table 3. Both di- and tri-substituted compounds had acceptable recoveries from sea water (>83%). Recovery of MBT is reduced (58%) and is attributed to increased evaporative losses in the injection liner during solvent venting (MBT has a higher volatility than di- and tri-substituted organotin compounds). For measuring just MBT in coastal waters, a smaller injection volume (10 μL) at a slower injection speed (0.41 μL s^−1^) increases retention and gave a higher recovery (∼100%).

**Table 3:** Summary of performance data for PTV-LVI GC/MS-SIM method.

Compound	Natural conc. (ng L^−1^)	Measured conc. (ng L^−1^)	% Recovery	% RSD	LoD rounded (ng L^−1^)	LoQ rounded (ng L^−1^)
MBT	11.5	34.7	58	19	*	*
DBT	3.2	36.7	84	8	0.2	0.5
TBT	5.4	44.7	98	8	0.7	2.2
DPhT	ND	33.6	83	8	0.9	2.6
TPhT	ND	38.7	97	5	0.4	1.4

Degrees of freedom (n = 5), ND = not detected. * not measureable using a 50 μL injection.

An external proficiency scheme sample (RTC, Product ID QC1566, Lot LRAA2561, Sigma-Aldrich) containing TBT and TPhT in water is used to evaluate the performance of the method. The concentrations measured are in good agreement with the acceptance limits of the certified value (Table 4).

**Table 4:** Analysis of proficiency scheme sample for TBT and TPhT.

Analyte	Certified value (ng L^−1^)	Acceptance limits (ng L^−1^)	Measured concentration (ng L^−1^) (n = 3)
TBT	43.8 ± 1.5	24.1–63.5	43.0 ± 0.5
TPhT	33.0 ± 1.1	18.2–47.9	31.1 ± 1.0

## Additional information

Organotins are the most heavily used (estimated global consumption 40–80,000 t yr^−1^) organometallic compounds in the world; with uses including PVC stabilisers (DBT), chemical catalysts and precursors in glass coating (MBT) [6] and pesticides (TPhT) [7]. TBT is a legacy pollutant from its use as an anti-foulant, having a high toxicity and persistence in the aquatic environment. DBT and MBT are environmental degradation products of TBT. Since the banning of organotins in anti-foulant coatings, under the International Convention on the Control of Harmful Anti-fouling Systems on Ships (AFS Convention Annex 1, 2001) concentrations of TBT in coastal waters have declined [8], [9], [10]. Despite this decrease, concentrations in coastal waters can often exceed those harmful to aquatic biota (concentrations of TBT up to 10 ng L^−1^ in the UK typically) [9]. These values exceed the WFD environmental quality standards (EQS; maximum allowable concentration = 1.5 ng L^−1^ and annual average = 0.2 ng L^−1^ allowable concentrations of TBT). In order to achieve compliance with these EQS values, highly specialised, hyphenated instrumentation, such as GC-inductively coupled plasma-mass spectrometry [7], [8] and GC-tandem mass spectrometry [7], [11], [12] are required.

Currently, standard laboratory instrumentation (e.g. GC/MS and GC-flame ionisation or photometric detection) cannot meet the LoQ needed to detect organotins in coastal waters. In order to achieve these limits, additional time consuming and often costly, pre-concentration (solid-phase extraction, stir-bar sorptive extraction, or solid-phase microextraction) methods are needed [12], [13], [14]. The novel PTV-LVI GC/MS-SIM method described here, adaptable to most standard instruments, provides a simple, low-cost solution for the measurement of DBT, TBT, DPhT and TPhT at concentrations found typically in coastal waters worldwide.
